# Outcomes of radial head fractures treated with pre-curved metacarpal plate

**DOI:** 10.1186/s12891-023-06566-9

**Published:** 2023-05-30

**Authors:** Xiang Yang, Jiang Zhuang, Zhi Xiaosong, Wang Huasong

**Affiliations:** 1 Department of orthopedics, General Hospital of Central Theater Command, Wuhan, Hubei Province P. R. China; 2 Wuhan University of Science and Technology, Wuhan, Hubei Province P. R. China

**Keywords:** Pre-curved metacarpal plates, Radial head fracture, Open reduction and internal fixation, Mason II type, Mason III type

## Abstract

**Objective:**

To explore the clinical outcomes of MasonII/III radial head fractures without the neck involvement treated with pre-curved metacarpal plates.

**Methods:**

Ninety cases of Mason typeII/III radial head fractures without the neck involvement were retrospectively collected from the department of orthopaedics of our hospital from September 2015 to May 2021. Group A (n = 44) underwent open reduction and internal fixation with pre-curved metacarpal plate, and Group B (n = 46) were fixed by traditional T-shaped plates. The operation time and the incision length were recorded during the operation. The Mayo Elbow Performance Score (MEPS), Disability of Arm, Shoulder and Hand (DASH) score, visual analogue scale (VAS) for pain, range of motion (ROM) and post-operative complications were evaluated at the last follow-up.

**Results:**

All the patients were followed up for at least 12 months. There were no significant difference between two groups regarding operation time (54.2 ± 12.1 v.s 51.3 ± 7.2, mins), MEPS (88.9 ± 4.2 v.s 87.8 ± 4.4), DASH score (7.3 ± 4.6 v.s 9.0 ± 4.0), VAS (1.6 ± 0.8 v.s 1.7 ± 0.7), and ROM. However, the incision length was shorter in Group A (5.6 ± 0.5 v.s 6.6 ± 0.5, cm, *P* < 0.01). The postoperative complication rate was also lower in Group A (1/44 v.s 8/46, *P =* 0.02).

**Conclusion:**

Masson II/III radial head fractures without the neck involvement treated with pre-curved metacarpal plates could achieve satisfactory outcomes comparable to traditional T-shaped plates. Moreover, the invasiveness and postoperative complications are less in patients with pre-curved metacarpal plates.

**Level of evidence:**

III, retrospective comparison study.

## Introduction

Radial head fracture is a common elbow injury. The most common injury mechanism is a fall on the outstretched arm [1]. The fracture of the lateral radial head accompanied by cartilage injury of the humeral head usually occurs when the radial head collides with the humeral head under rotational or vertical violence [2]. Mason classification is commonly used to guide the treatment of radial head fractures. The optimal treatments of Mason II radial head fractures are still controversial. Mason II fractures are defined as displaced fractures (displacement, > 2 mm) of the radial head or neck without comminution, that are amenable to open reduction and internal fixation (ORIF) [3]. However, Mulders et al. reported that non-operatively treated adults with an isolated Mason II radial head fracture had similar functional results after one year compared to operatively treated patients [4]. For Mason III, the ORIF or arthroplasty is mostly recommended [5]. For the materials of internal fixation, the plates are widely used. However, enlarged incision easily damages the deep branch of the radial nerve during the placement of plates. And the plate is hard to be placed at the ideal position (i.e. the “safe zone” of the radial head) [6], which results in a high incidence of hardware failure and a decreased range of motion [7]. Headless compression cannulated screws have shown advantages in the fixation of simple intra-articular fracture fragment, but we found that it is challenging to fix comminuted or thin fracture fragment with screws, in which the screws are hard to maintain the mini-fragment stability or increase the incidence of iatrogenic fracture.

In this study, the low-profile straight metacarpal plate was innovatively applied in the treatment of radial head fracture. This nail & plate system simulates the “bamboo raft” mode, which assembles and fixes the comminuted fragments with moderate compression. This article retrospectively collected the cases of Mason II/III radial head fractures without the neck involvement using either this technique or traditional T-shaped plates from September 2015 to May 2021 in our hospital. The follow-up results of two groups were compared and analyzed. Our hypothesis was that the metacarpal plate technique was not inferior to the traditional anatomic T-plate technique.

## Materials and methods

### Study design and eligibility criteria

**Fig. 1:**
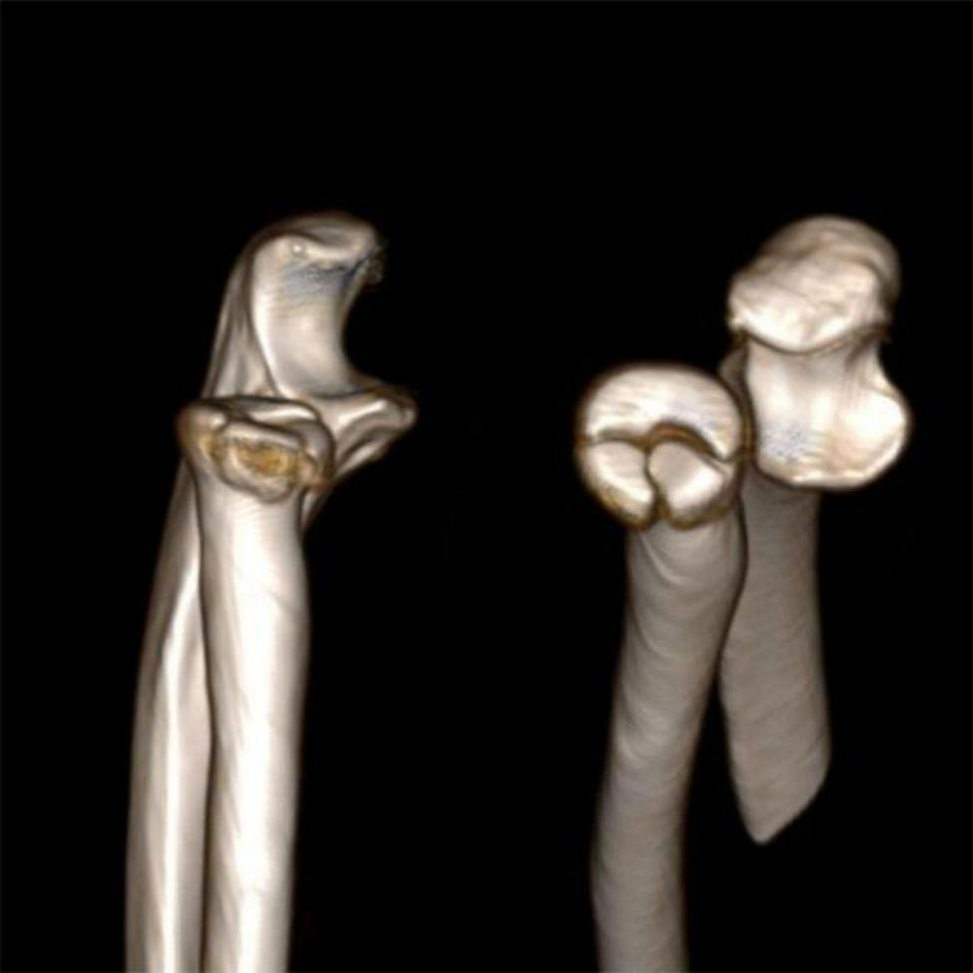
Three-dimensional computerized tomography reconstruction of the radial head fracture of one included case (Mason III)

The present study was a retrospective study of 90 consecutive patients diagnosed with Mason II or III radial head fracture, who underwent the open reduction and internal fixation using either pre-curved metacarpal plate (group A, n = 44) or T-shaped plate (group B, n = 46). Inclusion criteria: (1)over 18 years old; (2) patients with partial articular fracture of the radial head that had at least 2 mm articular step-off, as measured on an anteroposterior and lateral radiograph as well as Computed Tomography (CT)(Fig. 1); (3) follow-up of over 12 months. Exclusion criteria: (1) fractures of malunion already; (2) open or pathologic fractures; (3) accompanied by elbow dislocations or/and other concomitant elbow fractures; (4) previous elbow surgery, previous neurological disorders affecting the upper extremity; (5) fractures with the neck involvement or the radial head could not be reconstructed; (6) simple fractures which were well fixed with headless compression screws alone. The study was approved by the institutional ethical committee of our hospital.

### Operative technique

**Fig. 2:**
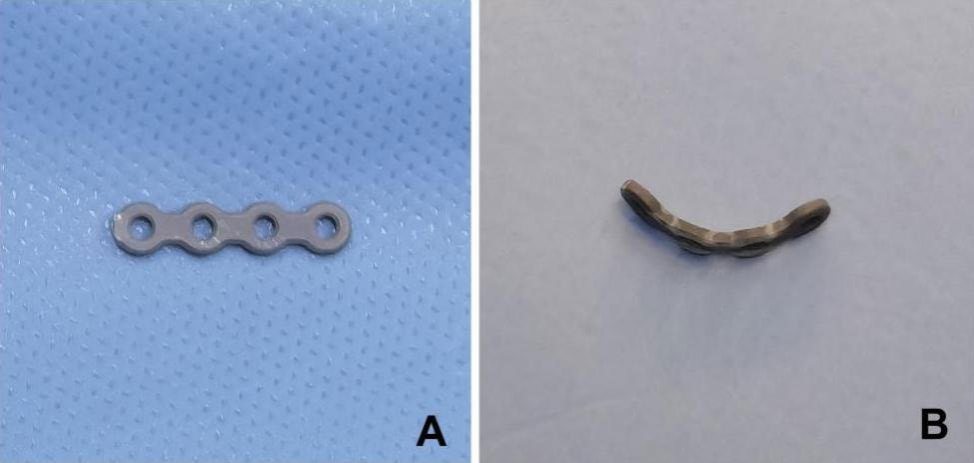
The metacarpal plate before and after bend. (A) straight metacarpal plate before bend, approximately 2.0 mm in length; (B) metacarpal plate after bend

**Fig. 3:**
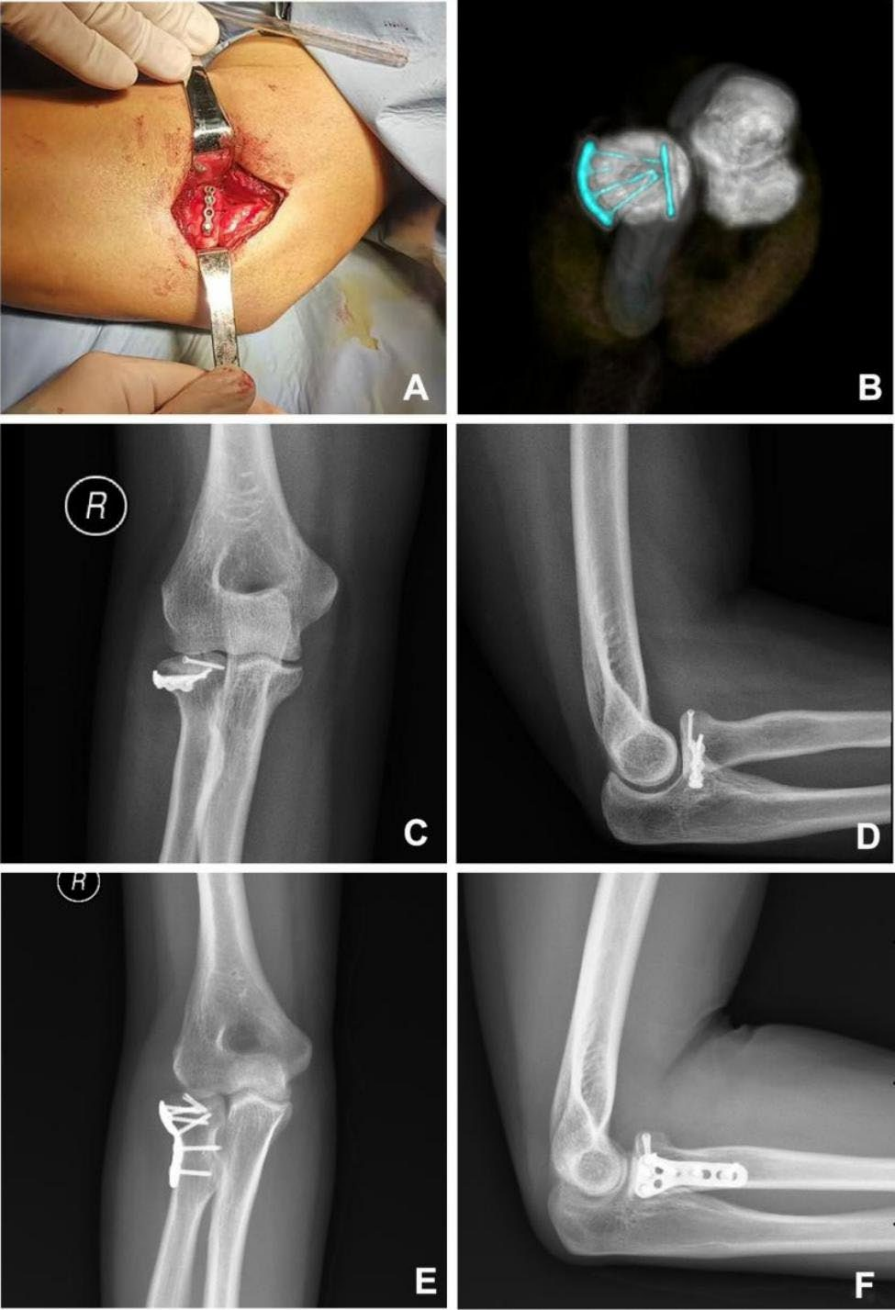
The placement of the pre-curved metacarpal plate and “T” plate. (A) Intraoperative image of Group A; (B) Postoperative three-dimensional computerized tomography of Group A; C, D. Anteroposterior and lateral radiograph of Group A; E, F. Anteroposterior and lateral radiograph of Group B

General anesthesia was applied in all patients. A tourniquet was used. All procedures were performed by one same certified senior orthopedic surgeon. The Kocher approach was used in all patients. The fracture fragments were reduced firstly. In Group A, the straight metacarpal locking plate (Tianjin ZhengTian Co., Ltd.) was broken off (4 holes, approximately 2.0 mm in length) and pre-curved according to the morphology of the lateral side of the radial head (“safe zone”) and to fit the surface (Fig. 2A, B), and then was placed where the longitudinal axis of the plate was parallel to the articular surface (Fig. 3A-D). In Group B, fractures were surgically fixed with the anatomic T-shaped locking plate (Tianjin ZhengTian Co., Ltd.) at the “safe zone” (Fig. 3E, F). For intra-articular fragments beyond the “safe zone” in two groups, headless compression screws (1.6 mm in diameter) were added for fixation. Annular ligament was repaired if possible.

### Postoperative management

The rehabilitation exercises were same for both groups. The elbow was supported with an adjustable brace in the 90-degree flexion position immediately after surgery and the isometric contraction of the muscle groups around the elbow started from the day after surgery. Active range of motion exercises with brace were permitted over one week. Partial weight-bearing and full range-of-motion exercises without brace were allowed after four weeks. Full weight-bearing was permitted after two months.

### Outcomes

The operation time, and the incision length were recorded during the operation. The Mayo Elbow Performance Score (MEPS), Disability of Arm, Shoulder and Hand (DASH) score, visual analogue scale (VAS) for pain, range of motion (ROM, including deficit in extention, flexion, pronation, supination) and post-operative complications were evaluated in postoperative months 3, 6, 12, 18 and continuing every 6 months.

### Statistical analysis

Data analysis was performed using GraphPad Prism 8. The continuous variables were reported as the means and standard deviations (Mean ± SD). The statistical power was set at 0.8, and the α level was 0.05. A post hoc power analysis was performed based on the postoperative DASH score. According to the results of the preliminary study (Group A, n = 15, 9.0 ± 3.1 versus Group B, n = 15, 10.4 ± 1.3), a sample size of at least 31 patients in each group would be required to identify a difference in the DASH score at a power of 0.80. A Chi-square test was used to compare the categorical variables. A two-sample t test or two-sample Wilcoxon rank-sum test was used to compare the continuous variables between the groups. The significance level was set at 0.05.

## Results

There was no significant difference between the two groups regarding age (n.s), gender (n.s), injury side (n.s), fracture type (n.s), and follow-up time (n.s) (Table 1).

**Table 1:** Characteristics of the included patients

	Group A	Group B	P value
Age(years)	38.0 ± 11.9	43.2 ± 13.5	n.s
Gender(n) male female	34 10	33 13	n.s
Injury side(n) left right	22 22	23 23	n.s
Type(n) MasonII MasonIII	33 11	32 14	n.s
Follow up(months)	18.5 ± 6.9	23.6 ± 16.1	n.s

**Fig. 4:**
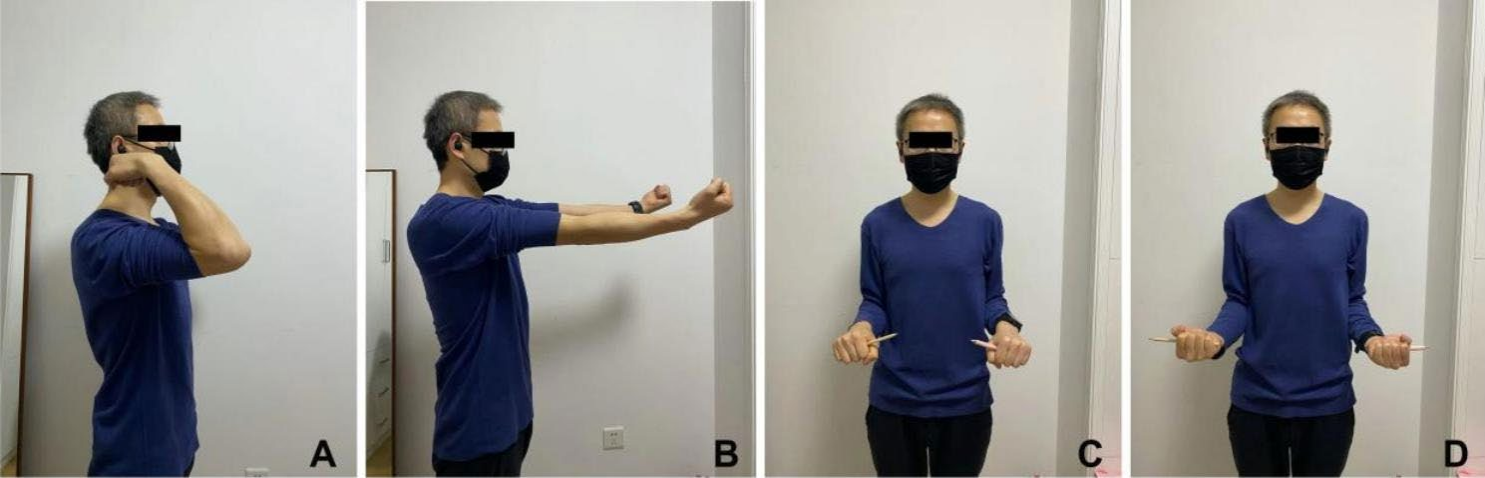
The functional images of a 41-year-old man at one year after surgery in Group (A) A. flexion; (B) extension; (C) pronation; (D) supination. Ipsilateral side (right), contralateral side (left)

There was no significant difference between two groups regarding the operation time. However, the incision length was shorter in Group A (p < 0.01). For the postoperative evaluation, there were no significant difference between two groups with regard to MEPS, DASH, and VAS (pain). For the ROM, there was a trend toward reduced ROM in group B, but the differences between two groups were not statistically significant. In Group A, one patient had the elbow stiffness. In Group B, the postoperative complications included elbow stiffness (n = 3), heterotopic ossification (n = 1) and local pain or discomfort (n = 4), and four patients underwent removal of symptomatic hardware. Overall, the complication rate was lower in Group A (Fig. 4; Table 2).

**Table 2:** Intraoperative and postoperative outcomes of two groups

	Group A	Group B	P value, significance
Operation time(mins)	54.2 ± 12.1	51.3 ± 7.2	0.18, n.s
Mayo	88.9 ± 4.2	87.8 ± 4.4	0.23, n.s
DASH	7.3 ± 4.6	9.0 ± 4.0	0.07, n.s
VAS for pain	1.6 ± 0.8	1.7 ± 0.7	0.73, n.s
Incision length(cm)	5.6 ± 0.5	6.6 ± 0.5	0.00, *
Deficit in extention(°)	5.4 ± 1.8	6.6 ± 4.7	0.11, n.s
Flexion(°)	124.6 ± 11.5	119.1 ± 15.5	0.06, n.s
Pronation(°)	71.2 ± 8.0	68.5 ± 10.3	0.16, n.s
Supination(°)	67.6 ± 6.7	63.3 ± 15.0	0.09, n.s
Complications stiffness heterotopic ossification local pain/discomfort	1/44 1/44	8/46 3/46 1/46 4/46	0.02, *

*, significance; n.s, no significance

## Discussion

In this study, we described an alternative fixation system (i.e. pre-curved metacarpal plate) to the traditional anatomic plate to treatment MasonII/III radial head fractures without the neck involvement, which was especially recommended when intra-articular fragments were thin or small and were hard to be fixed with headless compression screws. We found this fixation system could achieve comparative favorable outcome to traditional anatomic plate, and the invasiveness and postoperative complications were less in patients with pre-curved metacarpal plates.

The appropriate management of radial head fractures is key for successful outcomes and the avoidance of complications, including post-traumatic arthritis, stiffness, and instability. At present, anatomic reduction of the articular fracture of the radial head is critical for early recovery especially when the displacement is > 2 mm with loss of articular congruity [8]. Headless compression cannulated screws were widely used in Mason II fractures. However, we found that the fracture block was hard to be fixed with screws alone when it was very thin or small, and even worse, it might lead to secondary fracture, in which the (addition of) plates were needed. Mason III fractures usually require surgical intervention, including radial head resection, ORIF, radial head prosthesis replacement. For transverse radial head and neck fractures, anatomic buttress plates were commonly used. Recently, the “tripod technique” using headless compression screws for both articular and extra-articular transverse radial head and neck fractures was described as an alternative method [9, 10].

For comminuted intra-articular fractures without the neck involvement, ORIF is challenging. Screws alone are indicated for simple fractures when the fragments are easy to reduce and fix. However, screws may lead to nonunion and movement disorders result from insufficient tension and buttress. Historically, traditional anatomic proximal radius plate (usually “T” or “L” shaped plates) are mostly used for fixation [5, 11]. However, the obvious advantages of the plates are mainly manifested in the fixation for simple radial head-neck fractures or axial instability. For comminuted fractures, it may not cover and hold all intra-articular fragments. Moreover, the placement of plates requires extensive soft tissue stripping, which increases the risk of nerve injury (posterior interosseous nerve), bone nonunion and postoperative synarthrophysis [12, 13]. If the plate is thick, the dissected annular ligament during exposure is hard to be repaired due to the excessive tension, which decreases the elbow stability [14, 15]. In addition, thick plates increase the incidence of local crepitation and pain during forearm rotation after operation.

In this study, the low-profile straight metacarpal plate was innovatively used to treat Mason II/III radial head fractures without the neck involvement. The screw & plate system simulates the “bamboo raft” mode, which assembles and fixes the comminuted fragments with moderate compression. The plate shape is adjustable, and can fit into the various radial heads well. Compared to the anatomic plate, the metacarpal plate is smaller (4 holes, approximately 2.0 mm in length) and thinner, which doesn’t need excessive incision and may decrease the incidence of nerve injury. Moreover, almost all annular ligament of metacarpal plate group could be repaired, which was also a superiority over anatomic plate. In this study, there was no significant difference regarding operation time between two groups. However, the incision length was dramatically smaller in Group A. In the follow up, the patients in Group A achieved satisfactory results comparable to that in Group B when evaluated by MEPS, DASH, VAS scales and ROM, and the results were similar to those of other studies [4, 11, 16, 17]. The common postoperative complications of ORIF include elbow stiffness, traumatic arthritis, heterotopic ossification, necrosis, bone ununion, broken/loosen of the implants, et al. This study showed that the total complication (stiffness, local pain/discomfort) rate was lower in Group A, which was probably attributed to the mini-invasiveness of this technique and the mini-size of this system.

For fractures with the neck involvement or axial instability, the mini T plate or locking proximal radius plate were recommended [9]. Interestingly, Gregori et al. recently reported a technique that the radial head was placed in its anatomic position after in vitro reconstruction, and no plate was used for fixation with the radial shaft. Compared with the traditional anatomic plate internal fixation, Mayo’s elbow function score was higher, although 70% of the cases developed nonunion [7]. For Mason III fractures with radial neck, whether the use of pre-curved metacarpal plate for sole reconstruction of radial head was reliable deserves further elucidation.

This study also has some limitations: (1) lack of long-term follow-up data; (2) It is a retrospective study with low level of evidence and a potential selection bias. In the future, long-term follow-up large sample size prospective cohort study (or randomized controlled study) will have more guiding significance.

## Conclusion

Masson II/III radial head fractures without the neck involvement treated by pre-curved metacarpal plate could achieve satisfactory clinical results comparable to traditional T-shaped plate. Moreover, the invasiveness and postoperative complications are less in patients with pre-curved metacarpal plate.

## Data Availability

The datasets used and/or analyzed during the current study are available from the corresponding author on reasonable request.
